# The OBS UK Dashboard: an interactive tool for representative trial site selection to facilitate equality and diversity in maternity research

**DOI:** 10.1186/s13063-024-08487-x

**Published:** 2024-09-27

**Authors:** Amy Elsmore, Tanvi Rai, Philip Pallmann, Julia Townson, Sarah Kotecha, Mairead Black, Julia Sanders, Rachel Collis, Peter Collins, Bala Karunakaran, Pensee Wu, Sarah Bell, William Parry-Smith

**Affiliations:** 1 The Shrewsbury and Telford Hospitals NHS Trust, Telford, UK; 2 School of Medicine, Keele University, Keele, UK; 3 Nuffield Department of Primary Care Health Sciences, University of Oxford, Oxford, UK; 4 Centre for Trials Research, Cardiff University, Cardiff, UK; 5 University of Aberdeen, Aberdeen, UK; 6 School of Healthcare Sciences, Cardiff University, Cardiff, UK; 7 Cardiff and Vale University Health Board, Cardiff, UK

**Keywords:** Equality and diversity in research, Trial site selection, Maternity care, Health inequalities, Index of Multiple Deprivation

## Abstract

**Background:**

Obstetric Bleeding Study UK (OBS UK) (award ID: 152057) is a National Institute for Health and Care Research (NIHR)-funded stepped wedge cluster randomised controlled trial of a complex intervention for postpartum haemorrhage. This was developed in Wales and evaluated in a feasibility study, with improvements in maternal outcomes observed. Generalisability of the findings is limited by lack of control data and limited ethnic diversity in the Welsh obstetric patient population compared to the United Kingdom (UK): 94% of the Welsh population identifies as White, versus 82% in the UK. Non-White ethnicity and socioeconomic deprivation are linked to increased risk of adverse maternal outcomes. Traditionally, decisions regarding site selection are based on desire to complete trials on target in ‘tried and tested’ research active institutions. To ensure widespread applicability of the results and investigate the impact of ethnicity and social deprivation on trial outcomes, maternity units were recruited that represent the ethnic diversity and social deprivation profiles of the UK.

**Method:**

Using routinely collected, publicly available data, an interactive dashboard was developed that demonstrates the demographics of the population served by each maternity unit in the UK, to inform site recruitment. Data on births per year, ethnic and socioeconomic group of the population for each maternity unit, across the UK, were integrated into the dashboard.

**Results:**

The dashboard demonstrates that OBS UK trial sites reflect the ethnic and socioeconomic diversity of the UK (study vs UK population ethnicity: White 79.2% vs 81.7%, Asian 10.5% vs 9.3%, Black 4.7% vs 4.0%, Mixed 2.5% vs 2.9%, Other 3.0% vs 2.1%) with variation in site demography, size and location. Missing data varied across sites and nations and is presented.

**Conclusion:**

The NIHR equality, diversity and inclusion strategy states studies must widen participation, facilitating individuals from all backgrounds to engage. The development of this novel interactive dashboard demonstrates an innovative way of achieving this. National Health Service (NHS) maternity researchers should consider using this tool to enhance diversity in research, address health disparities and improve generalisability of findings. This approach could be applied to healthcare settings beyond maternity care and across different global populations.

**Trial registration:**

ISRCTN 17679951. Registered on August 30, 2023.

**Supplementary Information:**

The online version contains supplementary material available at 10.1186/s13063-024-08487-x.

## Background

Research trials often lack recruitment from communities worst affected by disease [1] and frequently take place in predominantly well-resourced academically linked centres. In the United Kingdom (UK), the National Institute for Health and Care Research (NIHR) has attempted to address this by developing ‘heat maps’ demonstrating the imbalance between research activity and areas of high disease burden [2, 3]. Rai et al. [4] found that chief investigators most often selected trial sites with well-established research track records and/or where they had existing relationships with local staff. This research culture propagates biased research and risks reproducing or exacerbating existing health inequalities [5]. Inclusion of a diverse population to trials is essential for generalisable and robust science that produces results that are applicable to the whole affected population. This is a major focus for patient and public involvement (PPI) groups, national bodies and funders of research and is reflected in the NIHR equality, diversity and inclusion strategy [6] and equivalent research agendas in other high-income countries [7, 8].

Despite having one of the lowest maternal mortality rates in the world, significant disparities in maternal and foetal outcomes exist in the United Kingdom (UK) for women of different ethnic and socioeconomic groups [9]. Women from Black and South Asian backgrounds are more likely to experience morbidity during childbirth, such as postpartum haemorrhage (PPH) [10], which persists when adjusted for maternal, foetal and birth characteristics. Black women in the UK also have increased risk of admission to intensive care after childbirth compared to women from other ethnic groups (adjusted odds ratio (OR) 1.69, 95% confidence interval (CI) 1.37 to 2.09) [11, 12]. The 2023 Mothers and Babies: Reducing Risk through Audits and Confidential Enquiries across the UK (MBRRACE-UK) report [13] found that maternal mortality for Black women is four times and for Asian women twice that for White women, during or up to 6 weeks after the end of their pregnancy. These ethnic disparities have been evident since the incorporation of ethnicity data collection in 2000 [14]. Similarly in the United States, Black women are at increased risk of severe morbidity and mortality compared to White women [15, 16]. Socioeconomic deprivation is also linked to increased risk of adverse pregnancy outcomes with women living in the most deprived areas of the UK twice as likely to die than the least deprived between 2019 and 2021 [13]. The significant overlap between minority ethnicity [13, 14] and socioeconomic deprivation in the UK means that these factors (and potential others including disability, migration status and English language competency) interact to compound disadvantage, contributing to poor maternity outcomes. Patient advocacy groups have expressed concern at these disparities [17].

The Obstetric Bleeding Study UK (OBS UK) is a NIHR-funded trial of a maternity care complex intervention for PPH. The care bundle was developed in Wales and rolled out in all Welsh National Health Service (NHS, the UK’s public healthcare system) maternity services as a quality improvement programme and feasibility study (OBS Cymru) [18]. Substantial reductions in PPH and red blood cell transfusion were reported [19]. Evidence of intervention effectiveness was, however, limited by the lack of randomisation or control data, and the paucity of ethnic diversity in the Welsh maternal population limited its generalisability to the wider UK setting. In Wales, 94% of the population identify as White versus 82% in the UK population (England and Wales, Census 2021) [20].

The OBS UK intervention will be delivered and evaluated at the maternity unit level, including all women giving birth under the care of participating maternity services. Recruitment at unit level will reduce socioeconomic and ethnic biases common in studies recruiting individual participants. A key goal of the trial is to investigate the role of ethnicity and social deprivation on the implementation of the intervention, as well as on clinical, economic and psychological outcomes. This requires recruitment of maternity units that reflect the ethnic and socioeconomic diversity of the whole UK population.

To achieve this, sociodemographic data from publicly available sources for the populations served by maternity services was accessed to create an interactive dashboard. This information was used to inform representative site selection in OBS UK from a larger number of interested candidate sites.

## Methods

### Data sources and collection

The most recent, publicly available, routinely collected data were used to ascertain area level information regarding the population served by all NHS Trusts or Health Boards (organisational units within the NHS, responsible for managing hospitals, community services and other healthcare facilities within a geographical area), for each nation in the UK (England, Scotland, Wales, Northern Ireland).

For ethnic groupings, data were collected into five aggregated groups as used by Office for National Statistics (ONS): White, Asian, Black, Mixed and Other.

Information regarding socioeconomic group was obtained from the Index of Multiple Deprivation scores (IMD). IMD datasets are small area measures of relative deprivation across each of the nations of the UK. Areas are ranked from most to least deprived. This measure is derived and presented differently in each nation.

#### England

Data were obtained regarding number of births per year in England as a whole and for each NHS Trust offering maternity care, from 01/04/2021 to 31/03/2022. These data were sourced from Hospital Episode Statistics (HES) via NHS Digital (the health and data technology agency for the NHS in England) [21]. HES is a reliable administrative dataset which captures admissions at NHS hospitals in England [22]. Birth rate data for one Trust (East Cheshire NHS Trust) were obtained from their own maternity services website [23], as it was not available through HES.

The National Maternity Dashboard [24] provides descriptive statistics and demographic data, sourced from the Maternity Services Data Set (MSDS), which provides a profile of the maternity population and activity for Trusts in England. The MSDS is an administrative dataset used by providers of maternity care in England for clinical purposes. ‘Ethnicity of mother at booking’ and ‘IMD of mother at booking’ were extracted for all Trusts offering maternity services in England. These data pertain to September 2022, as they were the most recent data available at the time of collection. IMD in England comprises of seven domains, which combine to create the IMD [25].

#### Scotland

Births per year for Scotland were obtained from Public Health Scotland (PHS) [26], from 01/04/2021 to 31/03/2022. These data were available per Scottish NHS Health Board. Maternal ethnicity was available via Scottish Morbidity Record 02 (SMR02) [27].

The Scottish IMD 2020 (SIMD) [28] is measured across seven domains but is evaluated differently than in England and is only available for the whole population (not maternity specific). Therefore, as a proxy for SIMD in the maternity population, data were compiled for the percentage of females per Health Board aged between 16 and 49 years in each SIMD decile [29]. This population level approach has been used for producing ethnicity data in a previous study [22].

#### Wales

Births per year in Wales were compiled per Health Board using data sourced from the Maternity Indicators (MI) dataset [30]. These birth rate data are from 2021. Ethnicity data on the Welsh maternity population as a whole [31] and per Health Board [32] were available from StatsWales from 2022, the latest available at the time of data collection. The MI dataset in Wales does not provide any socioeconomic data; therefore, we have used population level IMD data per Health Board. Similar (but different) to the English and Scottish versions, Welsh IMD (WIMD) is made up of eight separate domains of deprivation, but unavailable for specific analysis of females of reproductive age [33].

#### Northern Ireland

Births per year in Northern Ireland were obtained from the Northern Ireland Statistics and Research Agency (NISRA) [34], which was available by Health and Social Care Trust (health and social care provider in Northern Ireland, responsible for delivering services within a geographical area) for 2021. Ethnicity data were compiled for local government districts (a geographical area administered by a local government authority, providing services such as healthcare to the local population) from Census 2021 data [35]. Rather than the five aggregated groups used by ONS, these data are reported according to 12 different ethnic groups. We have therefore amalgamated them into the five ONS groups for ease of comparison.

Northern Ireland Multiple Deprivation Measure (NIMDM) was last updated in 2017 and ranks areas for seven domains of deprivation, combined to give an overall measure [36]. As for ethnicity, these deprivation data were only available per local government district.

### Creating the site selection tool—the OBS UK Dashboard

The raw data was entered into Microsoft Excel™ and tables and figures were developed. Subsequently, data were used to develop the site selection dashboard in Microsoft Power Bi™. Missing data were presented as such in the dashboard, and when being used to make decisions about site selection this missing data was excluded from the calculation of percentages (i.e. using the total number of non-missing data points as denominator), assuming that data was missing completely at random with the likelihood of a data point missing being the same across the different ethnic or socioeconomic groups.

### Using the dashboard for site selection in OBS UK

More than 50 sites expressed interest in taking part in the trial; after screening against eligibility criteria, 40 sites were eligible. Eligibility criteria included maternity units with > 2000 births per year and not currently using point-of-care coagulation testing on delivery suite. This excluded all sites in Wales where the intervention had already been fully embedded in routine care. If there was more than one maternity unit in a Trust or Health Board, only one unit was eligible to take part, due to the difficulties for randomisation this would pose and the potential for contamination across sites. The dashboard was then used to inform the selection of 36 sites for participation. Each site was evaluated based on its size (births per year), ethnicity and IMD data and grouped into broad categories based on these criteria, with the aim of achieving an overall study and site population that mirrored the diversity seen in the UK.

## Results

Figure 1 shows the OBS UK interactive dashboard, and Fig. 2 shows the selected OBS UK trial sites that are representative of the demographics of the UK population. An interactive version of Fig. 2 is available at https://obsuk.org [37]. The underlying data are shown in Supplementary Figs. 1–8 which present ethnicity and IMD data, across the UK.

**Fig. 1:**
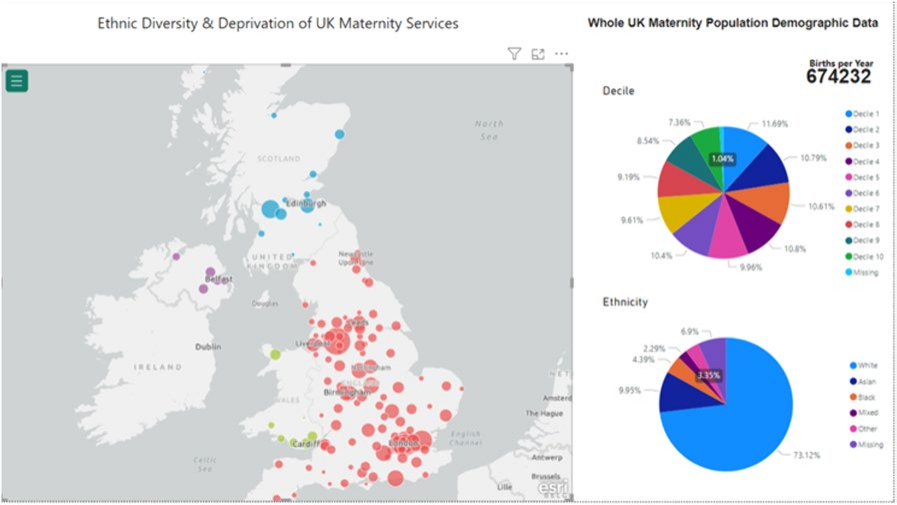
The OBS UK site selection dashboard

**Fig. 2:**
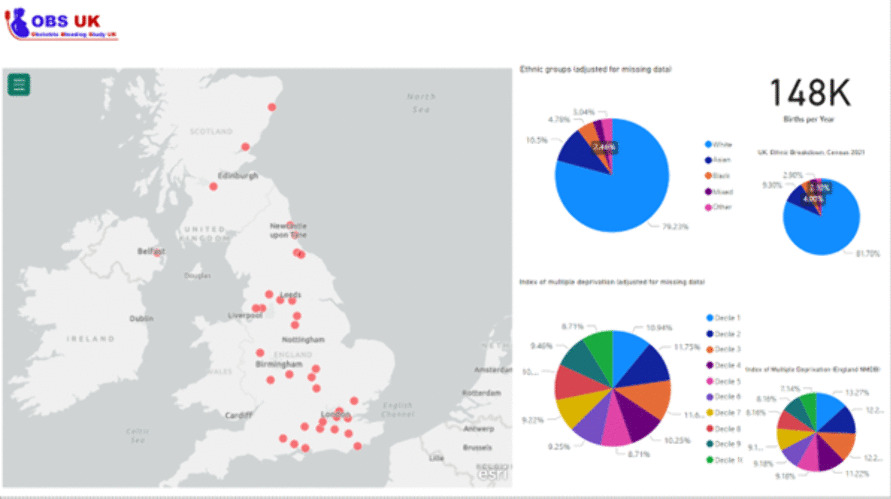
The OBS UK site selection dashboard—showing the 36 sites that have been enrolled onto the OBS UK trial

### The dashboard

Figure 1 demonstrates the interactive dashboard, in which all Trusts or Health Boards offering maternity services in the UK are represented. The size of the dot is proportional to the number of births per year.

Information within the screenshot shown is all data combined. Selecting a dot on the map displays data for that particular Trust/Health Board/Health and Social Care Trust.

Table 1 displays the characteristics of the 36 selected trial sites for OBS UK. Of the 40 eligible sites that expressed interest in participating in OBS UK, those excluded were, when adjusting for missing data, less diverse (> 80% population from White ethnic background) and were less deprived, with the proportion of women from IMD deciles 1–3 (most deprived), ranging from 19 to 55%. Data for these sites were complete for births per year, with missing data for ethnicity ranging between 0 and 7%, and ≤ 1% for IMD. Selected trial site demographics, adjusted for missing data, are shown in Supplementary Table S1.

**Table 1:** OBS UK recruited trial sites, displaying region, setting, birth rate data and ethnic diversity and deprivation

Site	Setting (rural/urban/combination)	Region	Birth rate (deliveries/year)	Ethnic group (%)						Index of multiple deprivation (%)										
				**White**	**Asian**	**Black**	**Mixed**	**Other**	**Missing**	**Decile 1**	**Decile 2**	**Decile 3**	**Decile 4**	**Decile 5**	**Decile 6**	**Decile 7**	**Decile 8**	**Decile 9**	**Decile 10**	**Missing**
Site 1	Urban	England	6340	46	11	19	8	9	8	6	23	24	13	10	8	5	3	3	2	2
Site 2	Urban	England	5750	52	17	5	2	6	14	23	19	16	11	6	3	4	5	5	5	1
Site 3	Urban	England	3590	61	19	13	4	3	0	9	6	6	9	8	5	18	14	14	8	1
Site 4	Combination	England	5895	62	32	1	1	2	2	33	15	16	8	6	3	6	8	5	1	1
Site 5	Urban	England	5555	62	10	15	3	9	2	12	27	30	14	5	2	1	2	1	0	6
Site 6	Combination	England	3135	63	6	1	3	1	26	1	1	3	6	9	14	16	14	17	19	1
Site 7	Combination	England	4970	67	6	6	2	2	17	7	12	17	10	10	8	7	11	8	11	0
Site 8	Combination	England	4510	68	20	5	3	3	1	16	16	11	10	10	8	9	9	7	2	1
Site 9	Urban	England	3290	69	19	3	2	3	2	0	2	5	8	12	15	7	14	17	20	2
Site 10	Combination	England	4720	69	9	11	3	4	3	6	8	15	15	9	11	16	6	10	3	1
Site 11	Combination	Scotland	3405	69.9	2.6	0.2	0	1.6	25.9	11	9	11	8	8	11	14	12	11	5	0
Site 12	Combination	England	5680	72	22	2	2	1	0	13	19	17	12	10	6	10	7	5	2	1
Site 13	Combination	England	5270	74	15	5	2	1	4	0	1	4	13	10	16	10	13	17	16	0
Site 14	Urban	England	4095	75	10	6	4	2	2	11	14	12	5	8	6	8	11	16	9	1
Site 15	Combination	England	2455	77	14	5	2	2	2	19	9	14	9	5	7	7	7	2	2	19
Site 16	Combination	England	2865	77	9	2	4	4	7	0	2	2	4	9	7	9	20	20	30	0
Site 17	Combination	England	4630	78	5	1	3	9	2	38	8	4	5	8	7	8	11	7	4	0
Site 18	Combination	England	4800	80	6	4	1	1	7	6	9	12	12	9	12	10	11	8	9	0
Site 19	Urban	England	5220	81	10	3	3	2	1	9	9	11	14	6	12	11	9	10	11	1
Site 20	Combination	England	3885	82	4	1	1	1	9	6	10	8	10	14	11	11	8	6	8	8
Site 21	Combination	England	2100	84	9	2	2	1	1	0	1	5	7	10	15	10	14	15	25	0
Site 22	Combination	England	3275	84	4	4	3	1	4	6	13	16	9	7	6	7	13	7	15	1
Site 23	Combination	England	2085	84	5	3	3	3	3	0	3	3	8	5	24	16	18	21	11	0
Site 24	Combination	England	2025	84	2	2	2	2	7	21	16	12	12	5	9	2	9	7	5	0
Site 25	Combination	Scotland	6304	84.2	2.9	0.7	0.4	0.4	11.4	11	15	13	12	9	7	9	10	10	3	0
Site 26	Combination	Scotland	5102	85.6	4.1	2.7	0.7	2.5	4.5	1	4	6	10	8	12	12	17	15	15	0
Site 27	Combination	England	5680	86	6	3	2	2	2	2	4	4	7	9	17	16	12	14	17	0
Site 28	Urban	England	3640	87	4	5	1	3	0	22	22	16	13	8	4	8	5	5	1	1
Site 29	Combination	England	2785	90	4	2	4	2	0	10	12	8	14	12	12	8	12	4	4	0
Site 30	Combination	England	3150	91	1	1	1	3	2	10	12	12	12	12	8	5	9	10	10	1
Site 31	Combination	England	2775	93	2	2	2	2	2	5	14	16	16	9	11	11	5	7	5	2
Site 32	Combination	Northern Ireland	3937	93.12	3.74	1.34	1.2	0.59	0	27	14	5	6	5	5	7	6	5	19	0
Site 33	Combination	England	4785	74	5	1	1	1	19	5	9	7	9	15	12	11	16	9	5	0
Site 34	Urban	England	3900	59	8	16	4	6	6	4	17	12	13	10	8	10	10	9	7	1
Site 35	Urban	England	4100	29	48	6	2	6	4	11	28	29	15	7	4	2	2	1	0	0
Site 36	Combination	England	2515	87	4	4	2	2	2	27	15	12	6	8	4	8	6	12	4	0

The 36 sites participating in the OBS UK trial are illustrated within the dashboard in Fig. 2, with comparison to national data for ethnicity and deprivation. It demonstrates that the sites are widely spread across the UK geographically, and the combined ethnic diversity of women giving birth at planned study sites is similar to that of the whole UK population, and the same is true for the distribution of IMD deciles (Fig. 2).

This demonstrates wide geographical spread of sites and a trial population that is comparable to the wider UK maternity population in terms of ethnicity and deprivation levels.

Supplementary Figures S1–S8 present ethnicity and IMD data across the UK.

### England

Ethnicity and IMD of women at booking, for all Trusts offering maternity services in England, are shown in Figs. S1 and S2. Ethnicity is displayed as a percentage with missing data also shown. IMD is reported as percentage per decile, with decile 1 representing the most deprived and decile 10 the least deprived section of the population.

### Scotland

Figure S3 demonstrates maternal ethnicity per NHS Health Board of residence in Scotland. There is a high proportion of missing data for some of the Scottish Health Boards, most noticeably NHS Borders, NHS Orkney, NHS Shetland and NHS Western Isles. The latter three have very low birth rates (131, 171 and 194 births respectively in the year 01/04/2021–31/03/2022), and within the source data these three Health Boards were combined as ‘Islands’, hence them appearing to have the same proportions in the figure. Figure S4 presents SIMD data of females ages 16–49, per Health Board, displayed as a percentage.

### Wales

Figures S5 and S6 demonstrate ethnicity and WIMD respectively. Maternal ethnicity is reported as live births to Welsh residents by ethnic group and Health Board providing the service. Maternal WIMD is not routinely collected in Wales and StatsWales stated to us that they do not currently plan to report this. WIMD of the population per Health Board has therefore been compiled as a proxy measure of maternal deprivation.

### Northern Ireland

Figures S7 and S8 present population level data, displayed per local government district from the 2021 Census. Information per Health and Social Care Trust was not available therefore this approach was felt to be the most feasible given our time constraints and need for the data to identify which sites to recruit to our study.

## Discussion

Routinely collected data for ethnicity and socioeconomic status were extracted to produce demographic profiles of the population served at each maternity unit in the UK. The findings were used to create an interactive map tool that can display these data collectively in one place, and this tool informed selection of a diverse range of sites for the OBS UK trial, aiming to achieve a study population that mirrors the diversity seen in the UK. This approach could be adopted by future studies, including in non-maternity settings and for research conducted in countries beyond the UK, with the aim of reducing the risk of site selection biases often found in randomised trials and promoting recruitment of sometimes underserved populations.

The limitations of this work arise from the variation in how the data of interest (specifically ethnicity and IMD) of the maternity population are collected and reported between the four nations of the UK. Ethnicity and IMD of women at booking are reported per Trust offering maternity care, in England, via the National Maternity Dashboard. Maternity services datasets also exist in Scotland, Wales and Northern Ireland; however, data drawn into these differ from those in England. For Scotland and Wales, ethnicity of women giving birth per Health Board is recorded, yet SIMD/WIMD is not. These data were gathered from whole population sources. For Northern Ireland, the maternity dataset is not publicly available. Since ethnicity and deprivation data per Trust were not available, whole population data per local government district were used. For future iterations of the dashboard, we intend to request access to this via Health Data Research Innovation Gateway [38].

Regarding deprivation indices, as each UK country has a different set of indicators, time periods, domains and domain weights, IMD data for the four nations are compiled and presented differently. It is therefore important to be mindful that direct comparisons should only be made for NHS Trusts/Health Boards within nations, and any comparisons between nations be interpreted with caution. For some Trusts and Health Boards, there was a high proportion of missing data which challenges the accuracy of the source data and therefore the completeness of the dashboard. This missingness was assumed to be equally likely across ethnic and socioeconomic groups, which may not have been the case, potentially introducing misclassification bias. Complete and accurate ethnicity data are crucial for identifying and addressing health disparities. Missing ethnicity data may limit generalisability of study findings to the broader population. Healthcare organisations and healthcare professionals should be encouraged to improve ethnicity data collection through education, training and enhancing their data collection methods, such as using self-identification and a culturally sensitive approach from those collecting data. In addition, standardising the reporting of ethnicity across different systems is crucial for improving data comparability and facilitating the analysis of health disparities. Ethnicity recording is mandatory within the Maternity Data Standard in England [39]. Overall, the trial sites are spread throughout the UK, however, the Southwest of England appears underrepresented, as many maternity units here have already taken up the OBS care bundle, and as such do not meet the inclusion criteria of the trial. Welsh maternity units have all embedded the OBS care bundle as part of OBS Cymru, excluding them from OBS UK. The exclusion criteria for trial sites for OBS UK may have also had the potential to affect the diversity of our trial population. For example, small units with annual number of births < 2000, and units that already have point-of-care coagulation testing in routine use in obstetric care, such as large urban maternity units serving ethnically diverse and deprived populations are all excluded. Excluding smaller, more rural units may introduce bias as it could exclude individuals with characteristics that may not be fully represented in larger units, as these units often represent unique populations with distinct demographics. Rural units may have different staffing levels and access to resources compared to larger, urban units. However, for the OBS UK trial, we were constrained by our inclusion criteria of units with > 2000 births per year which were chosen due to the need to recruit a sufficiently large trial population. That notwithstanding, the OBS care bundle has been previously implemented into all obstetric units in Wales which report between 500 and 6000 births per year, including units based in very rural areas [19]. Furthermore, there are some population characteristics that we specifically noted for our trial population, such as disability, not having English as a first language, migration status and others that are likely to contribute to patient outcomes. To our knowledge, these data are not routinely collected at a national level; therefore, we were unable to include these factors in our work. Despite these limitations, we believe our trial population closely represents the UK, as was shown in Fig. 2.

To our knowledge, this is the first time that routinely collected data from large administrative and maternity datasets have been collated for the four UK nations and used to inform site selection for a large maternity trial. Although there is widespread interest in using routinely collected data to explore inequalities in care received by different ethnic groups [40], the reliability of these data must be considered when using them to draw conclusions regarding care or make decisions about study site selection as we have done with our dashboard. Jardine et al. [22] found that administrative (i.e. HES) and maternity datasets demonstrated good agreement on aggregated ethnic group (White, Asian, Black, Mixed, Other) but there is a generally recognised need to improve accuracy of recording ethnicity [41], and particularly of those classified as belonging to Mixed or Other ethnic groups, where there is potential for misclassification bias. A significant proportion of the data were obtained from Census 2021 [20], which undergoes a quality assessment prior to publication to assess its accuracy [42] and achieved a 97% response rate from UK households. The reliability of the dashboard data will be further tested by conducting a multicentre service evaluation survey, in which our trial sites will provide their patients’ demographic data for comparison with the dashboard.

The benefit of using routinely collected, publicly available data was that it ensured this project was viable, cost-effective and robust. The alternative would have been primary data collection (for example approaching each maternity unit in the UK and requesting their equality and diversity data), which would have been logistically challenging and not possible within our time constraints. The presented approach utilises methodology that is open, reproducible and pragmatic and will allow interval updates of the database and map tool to be performed from the source data (e.g. every 6–12 months). This will allow the dashboard to remain relevant and be of optimal benefit to researchers that may wish to use this approach for their own work. Currently, the data used to populate the interactive site selection dashboard is updated manually by researchers. Collaboration with IT (information technology) professionals is being explored to automate the process.

Traditionally, decisions regarding site selection for trials have been based on a desire to complete a trial to time and target, in ‘tried and tested’ research active institutions with proven research experience [4]. Few studies have examined site recruitment; Gheorghe et al. [43] perceived a mismatch between factors considered desirable for site selection and what really motivates site selection. Although widespread clinical applicability of results of a trial to the affected population is desirable to researchers, recruitment of sites is often based on more pragmatic factors, for example units with proven and positive research reputations, and those that are geographically convenient and already known to trial investigators. Further challenges appear when there is unequal access to research support or resources. For example, MacLellan et al. [44] found that the recruitment of sites that represented populations most at risk was less well supported by Clinical Research Networks (CRNs). This dashboard presents a novel approach to target research to locations where there is greatest need, with aspirations to increase the validity and generalisability of trial results and reduce health inequalities.

To enhance the applicability of this dashboard, future work should explore how this site selection methodology can be adapted to diverse healthcare settings such as outside of maternity populations and non-UK contexts. NHS Digital collects ethnicity data as part of various national datasets such as HES [45] (capturing information on hospital admissions, including ethnicity) and the National Cancer Registration and Analysis Service (NCRAS) [46] which also collects ethnicity as well as information on cancer diagnoses and outcomes. Globally, ethnicity data are routinely collected in national healthcare datasets such as the US the Centers for Disease Control and Prevention (CDC) National Vital Statistics System [47], and adequate inclusion in research is part of their national commitment towards improving the health of minoritised groups and reducing disparities [7]. Similar databases exist in Canada (Statistics Canada [48]) and Australia (Australian Bureau of Statistics [49]), as well as in many European and Asian countries. However, there are exceptions such as France and Germany, where the collection of ethnicity data is restricted or prohibited; it can pose significant challenges for researchers seeking to recruit a diverse study population and understand health disparities [50]. Socioeconomic status and deprivation data are also routinely collected in many countries but once again, they use different measures and are national context-specific, which engenders similar limitations for comparability. It is important to note that differences in how ethnic and social deprivation categories are defined and measured can make it challenging to compare findings. We had this experience even across the devolved nations within the UK, and therefore advise significant caution when drawing any inferences across different national contexts.

The NIHR equality, diversity and inclusion strategy [6] states that researchers must widen access and participation, facilitating individuals from all backgrounds to engage in research, and that one of the key activities to support this is the development of innovative models that help meet the unique needs of the target population. By developing a novel methodology for site selection that ensures the trial population is representative of the maternity population of the UK, this ethos has been incorporated into the OBS UK trial from the outset.

## Conclusion

Routinely collected, publicly available population data can be collated to provide information about the demographics of the maternity population in the UK. These efforts can be operationalised to inform site selection that ensures the study population is representative of the affected population. This approach should be considered by other research groups, beyond maternity care and the UK, to address the mismatch between where research is conducted and where the highest burden of disease lies. This may go some way to addressing inequalities in care and outcomes that are seen in ethnic minority and underserved groups.

The terms woman/women/mother are used consistently throughout this paper pertaining to the primary person receiving maternity care and giving birth. We acknowledge that not all birthing people use these terms to describe themselves and we want to promote gender equality throughout to ensure respect for the unique psychological, physiological and social needs of each individual. We specifically acknowledge the transgender and non-binary experience of pregnancy and childbirth.

## Supplementary Information

Additional file 1: Fig. S1 Bar graph demonstrating ethnic group of mother at booking, for each maternity unit in England.

Additional file 2: Fig. S2 Bar graph demonstrating Index of Multiple Deprivation (IMD) of mother at booking, for each maternity unit in England.

Additional file 3: Fig. S3 Bar graph demonstrating maternal ethnicity per NHS Health Board of residence in Scotland.

Additional file 4: Fig. S4 Bar graph demonstrating SIMD of females age 16–49 years per NHS Health Board in Scotland.

Additional file 5: Fig. S5 Bar graph demonstrating live births to Welsh residents by ethnic groups and Health Board.

Additional file 6: Fig. S6 Bar graph demonstrating WIMD of the population residing in each Health Board.

Additional file 7: Fig. S7 Bar graph demonstrating Northern Irish whole population ethnic group breakdown per local government district.

Additional file 8: Fig. S8 Bar graph demonstrating Northern Irish Multiple Deprivation Measure (NIMDM) per local government district.

Additional file 9: Table S1 Ethnicity and IMD data per trial site, adjusted for missing data.

## Data Availability

The datasets utilised in this study to produce the dashboard are publicly available: UK Census 2021. Office of National Statistics. Available at: https://census.gov.uk/census-2021-results [20] NHS Maternity Annual Statistics 2021-22. Available at: https://app.powerbi.com/view?r=eyJrIjoiZDc1MzQ1OTgtNjQxYS00NmExLWExYzQtYjIxN2I3MTY0ZGNjIiwidCI6IjUwZjYwNzFmLWJiZmUtNDAxYS04ODAzLTY3Mzc0OGU2MjllMiIsImMiOjh9 [21] National Maternity Dashboard. Available at: https://digital.nhs.uk/data-and-information/data-collections-and-data-sets/data-sets/maternity-services-data-set/maternity-services-dashboard [24] The English Indices of Deprivation 2019 Infographic. Available at: https://assets.publishing.service.gov.uk/government/uploads/system/uploads/attachment_data/file/833959/IoD2019_Infographic.pdf [25] Births in Scotland. Public Health Scotland. Available at: https://publichealthscotland.scot/publications/births-in-scotland/births-in-scotland-year-ending-31-march-2022/ [26] Scottish Morbidity Record 02, Information Services Division of NHS Scotland. Available at: https://www.ndc.scot.nhs.uk/Data-Dictionary/SMR-Datasets/SMR02-Maternity-Inpatient-and-Day-Case/ [27] Population Estimates by Scottish Index of Multiple Deprivation. Latest update Sept 2020. National Records of Scotland. Available at: https://www.nrscotland.gov.uk/statistics-and-data/statistics/statistics-by-theme/population/population-estimates/2011-based-special-area-population-estimates/population-estimates-by-simd-2016 [29] Maternity and birth statistics: 2021. Welsh Government. Available at: https://www.gov.wales/maternity-and-birth-statistics-2021-html#:~:text=Three%20in%20ten%20(29%25),for%20mothers%20birthing%20in%202021 [30] Live births to Welsh residents by ethnic groups and health board providing this service. Available at: https://statswales.gov.wales/Catalogue/Health-and-Social-Care/NHS-Primary-and-Community-Activity/Maternity/livebirthstowelshresidents-by-ethnicgroup-healthboardprovidingtheservice [32] Welsh Index of Multiple Deprivation (WIMD) 2019 Interactive Tool. Welsh Government. Available at: https://wimd.gov.wales/geography/lhb/W11000023?lang=en#&min=0&max=10&domain=overall [33] Birth Statistics. Northern Ireland Statistics & Research Agency. Available at: https://www.nisra.gov.uk/publications/birth-statistics [34] Census 2021 main statistics ethnicity tables. Northern Ireland Statistics & Research Agency. Available at: https://www.nisra.gov.uk/publications/census-2021-main-statistics-ethnicity-tables [35] Northern Ireland Multiple Deprivation Measure 2017 (NIMDM2017). Northern Ireland Statistics & Research Agency. Available at: https://www.nisra.gov.uk/statistics/deprivation/northern-ireland-multiple-deprivation-measure-2017-nimdm2017 [36]

## Abbreviations

CDC: Centers for Disease Control and Prevention
CI: Confidence interval
CRN: Clinical Research Network
HES: Hospital Episode Statistics
IMD: Index of Multiple Deprivation
IT: Information technology
MBRRACE-UK: Mothers and Babies: Reducing Risk through Audits and Confidential Enquiries across the UK
MI: Maternity Indicators
MSDS: Maternity Services Data Set
NCRAS: National Cancer Registration and Analysis Service
NHS: National Health Service
NIHR: National Institute for Health and Care Excellence
NIMDM: Northern Ireland Multiple Deprivation Measure
NISRA: Northern Ireland Statistics and Research Agency
OBS UK: Obstetric Bleeding Study UK
ONS: Office for National Statistics
OR: Odds ratio
PHS: Public Health Scotland
PPH: Postpartum haemorrhage
SIMD: Scottish Index of Multiple Deprivation
SMR02: Scottish Morbidity Record 02
UK: United Kingdom
US: United States
WIMD: Welsh Index of Multiple Deprivation
