# Supplementary figures and images for: The OBS UK Dashboard: an interactive tool for representative trial site selection to facilitate equality and diversity in maternity research
Source: Trials. 2024 Sep 27;25:629. doi: 10.1186/s13063-024-08487-x (PMC11429411; doi:10.1186/s13063-024-08487-x)

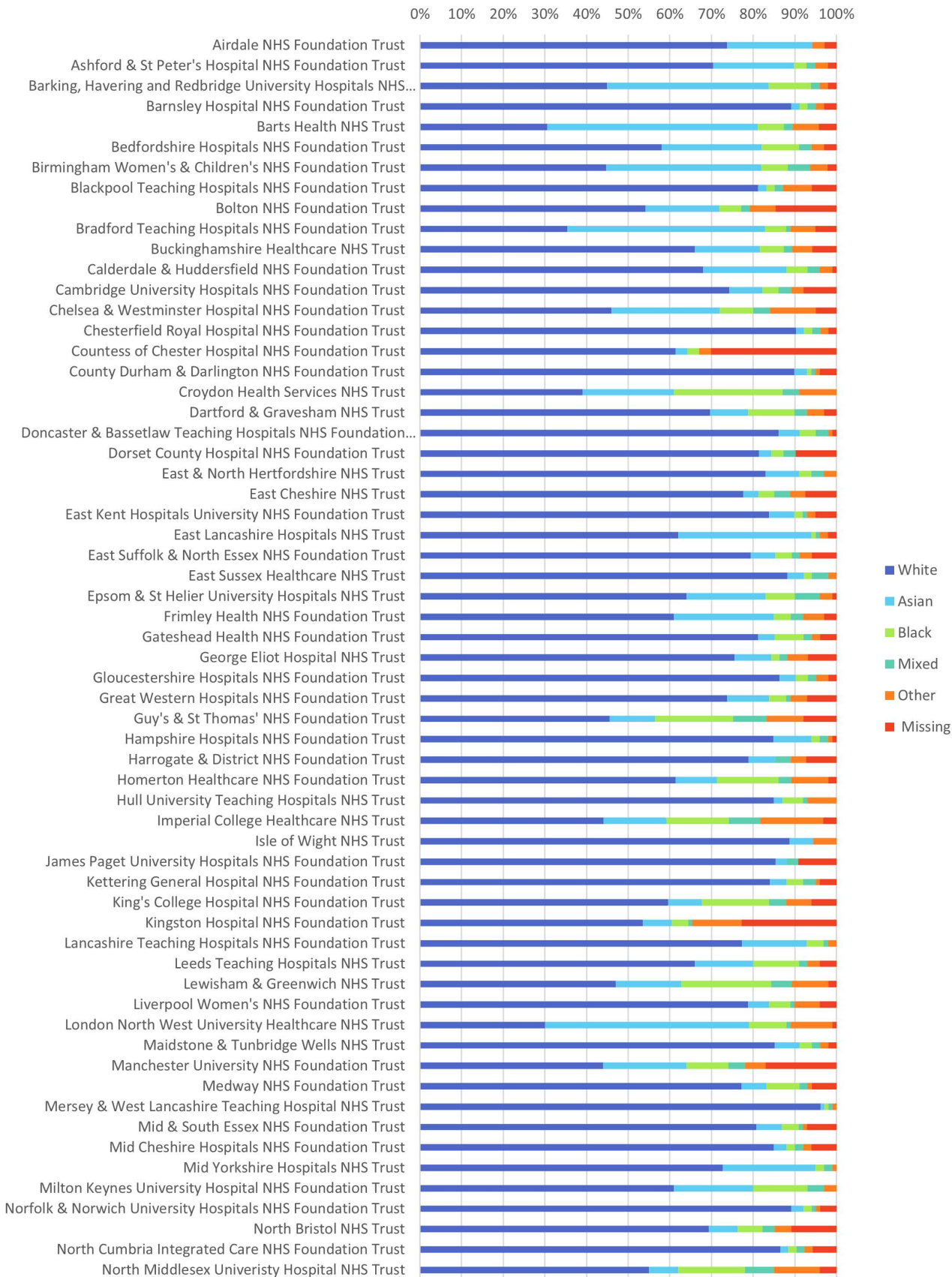

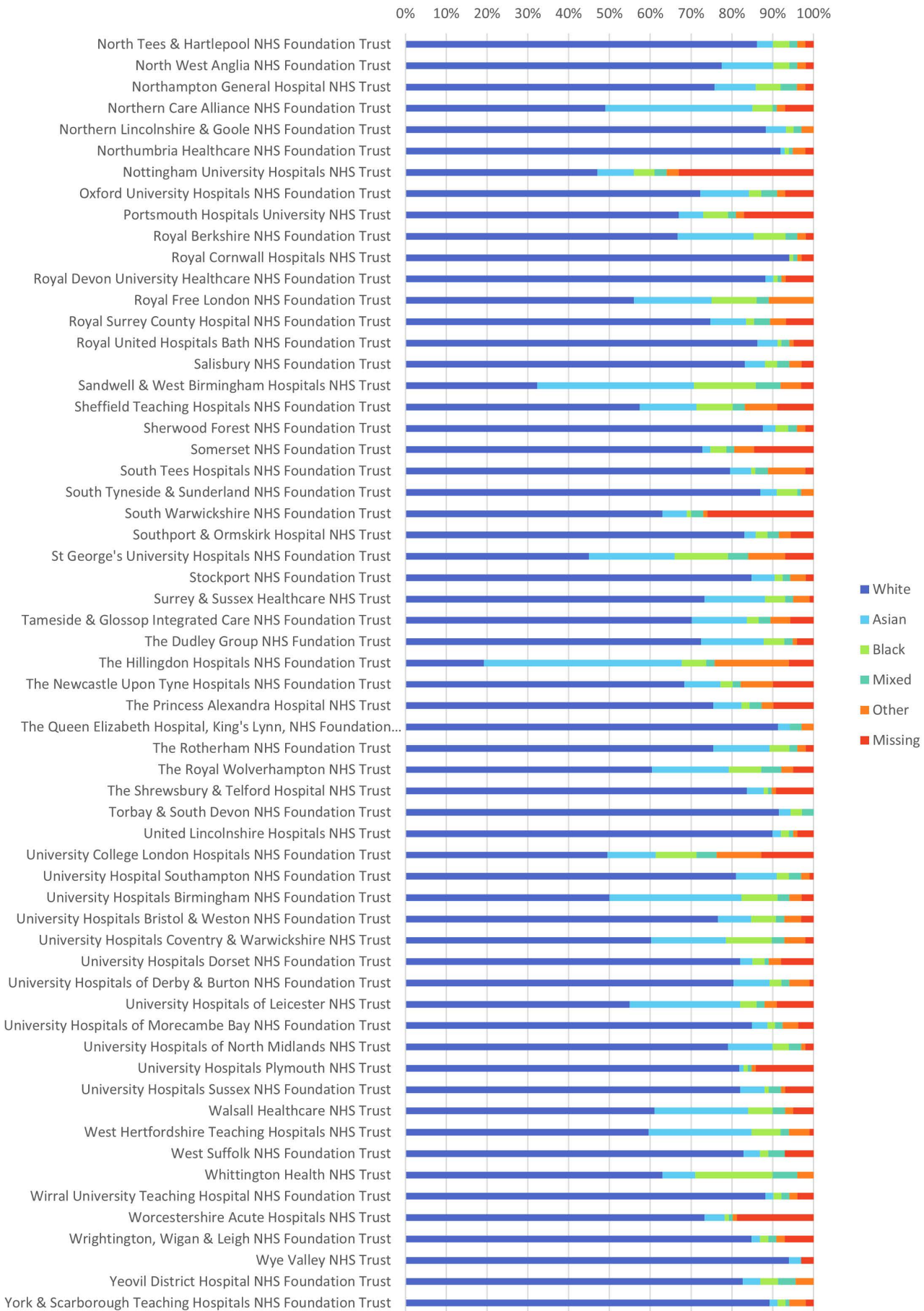

Supplement: Supplementary file 1 — Additional file 1: Fig. S1 Bar graph demonstrating ethnic group of mother at booking, for each maternity unit in England. [file 13063_2024_8487_MOESM1_ESM.pdf]

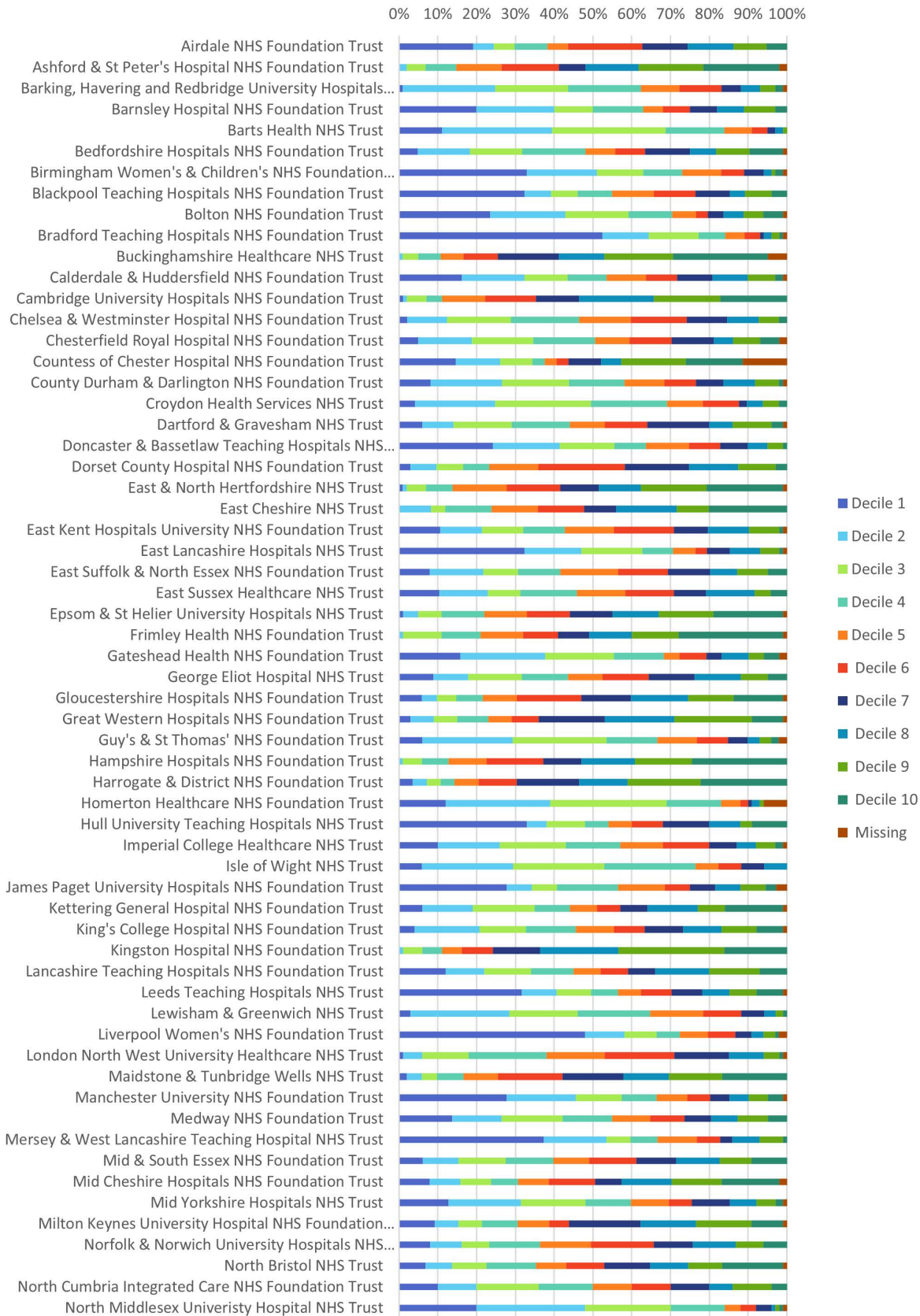

0% 10% 20% 30% 40% 50% 60% 70% 80% 90% 100%

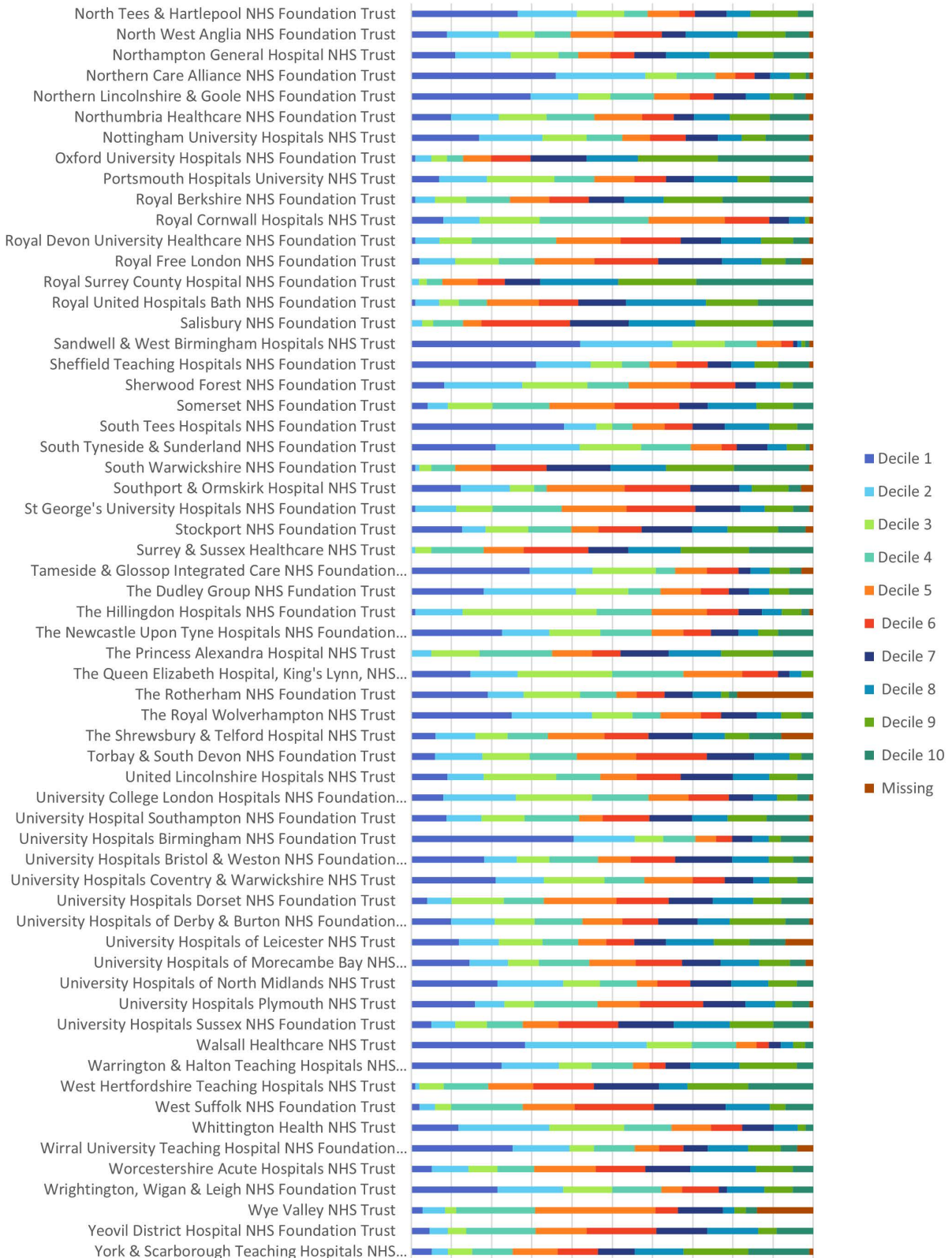

Supplement: Supplementary file 2 — Additional file 2: Fig. S2 Bar graph demonstrating Index of Multiple Deprivation (IMD) of mother at booking, for each maternity unit in England. [file 13063_2024_8487_MOESM2_ESM.pdf]

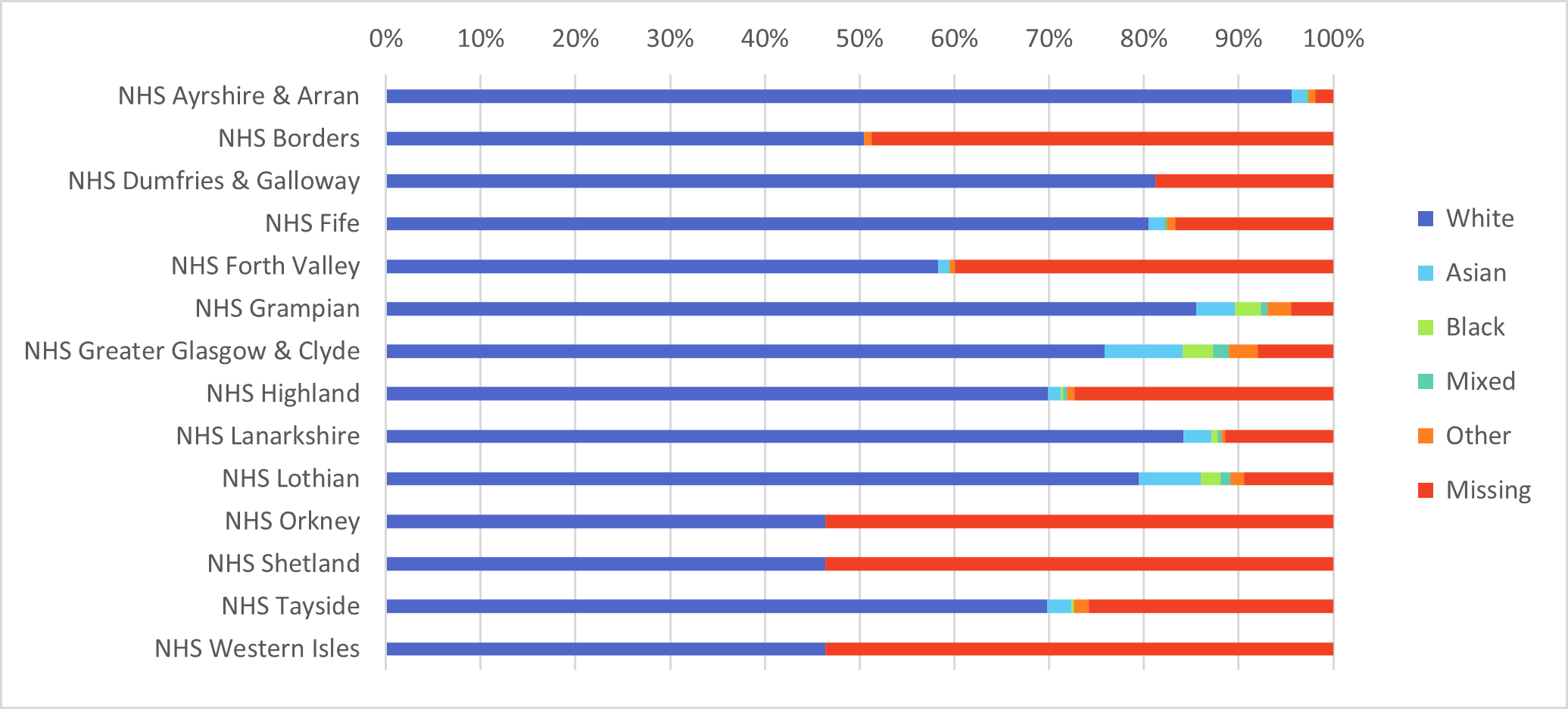

Supplement: Supplementary file 3 — Additional file 3: Fig. S3 Bar graph demonstrating maternal ethnicity per NHS Health Board of residence in Scotland. [file 13063_2024_8487_MOESM3_ESM.png]

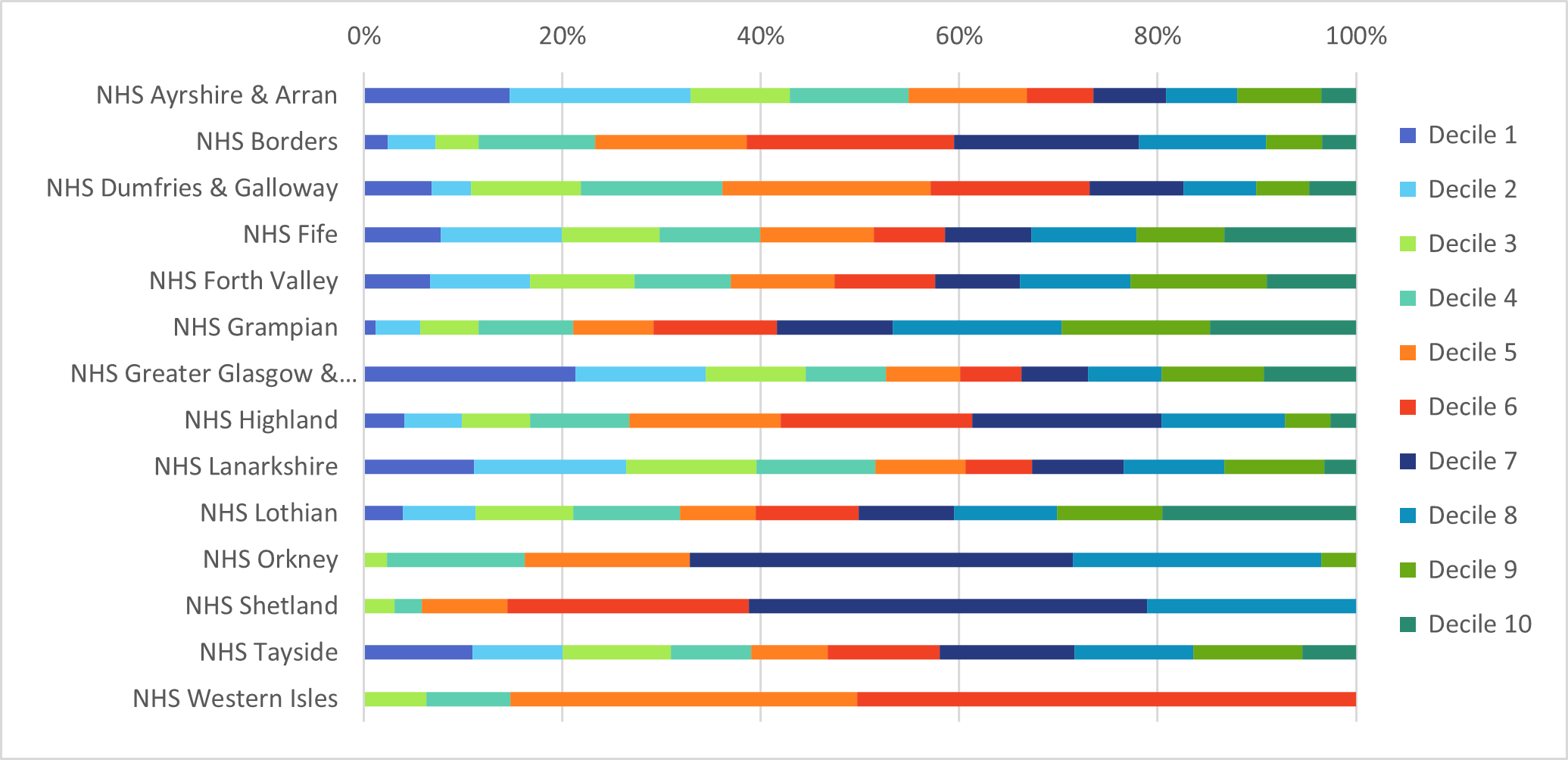

Supplement: Supplementary file 4 — Additional file 4: Fig. S4 Bar graph demonstrating SIMD of females age 16–49 years per NHS Health Board in Scotland. [file 13063_2024_8487_MOESM4_ESM.png]

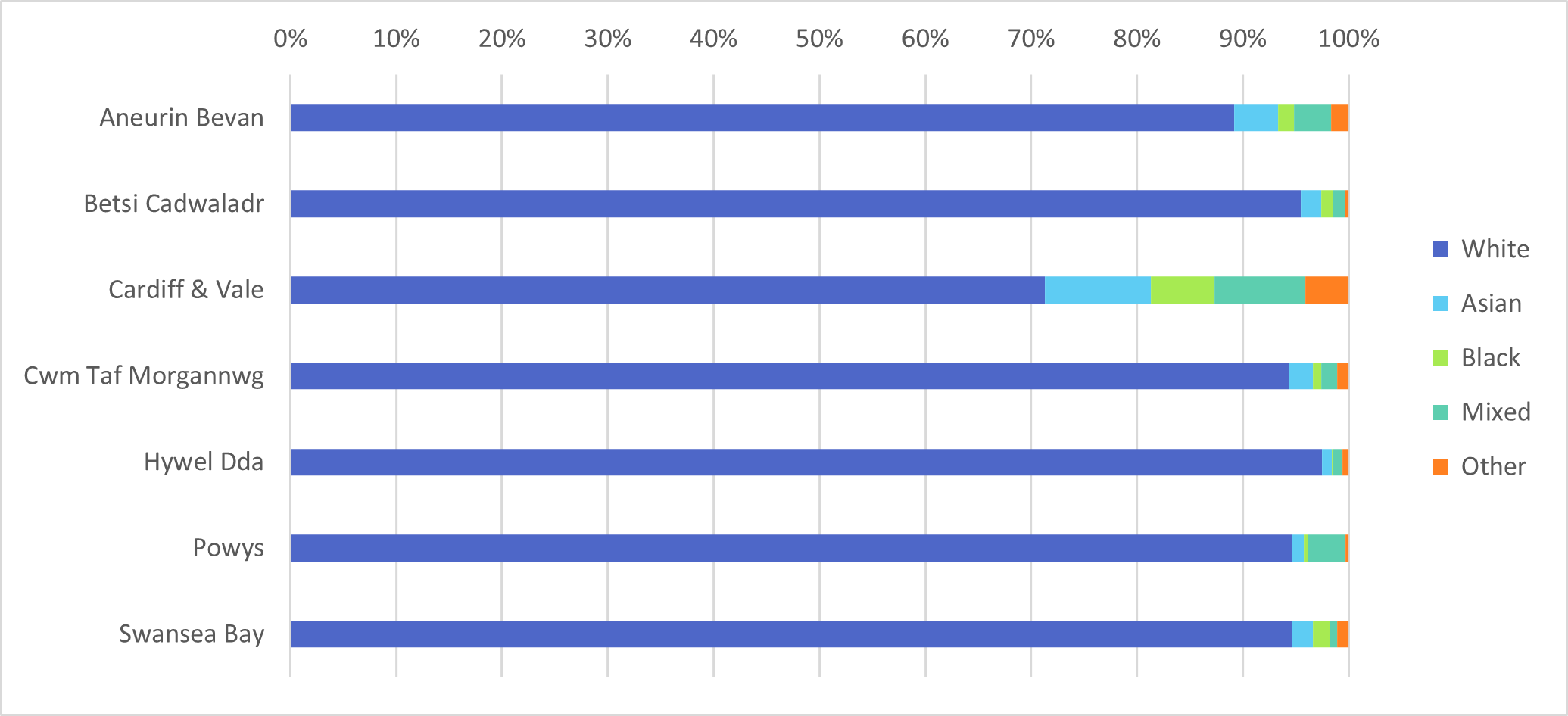

Supplement: Supplementary file 5 — Additional file 5: Fig. S5 Bar graph demonstrating live births to Welsh residents by ethnic groups and Health Board. [file 13063_2024_8487_MOESM5_ESM.png]

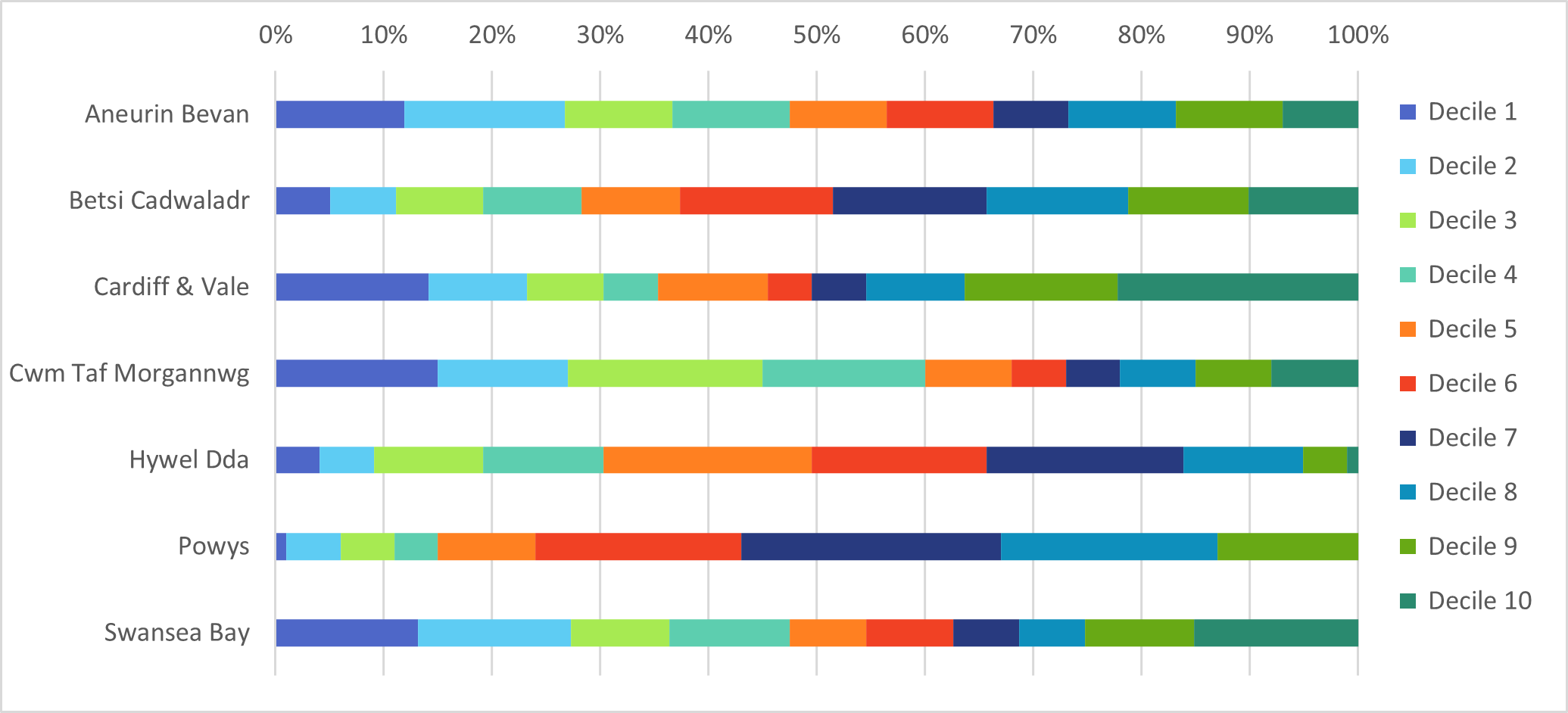

Supplement: Supplementary file 6 — Additional file 6: Fig. S6 Bar graph demonstrating WIMD of the population residing in each Health Board. [file 13063_2024_8487_MOESM6_ESM.png]

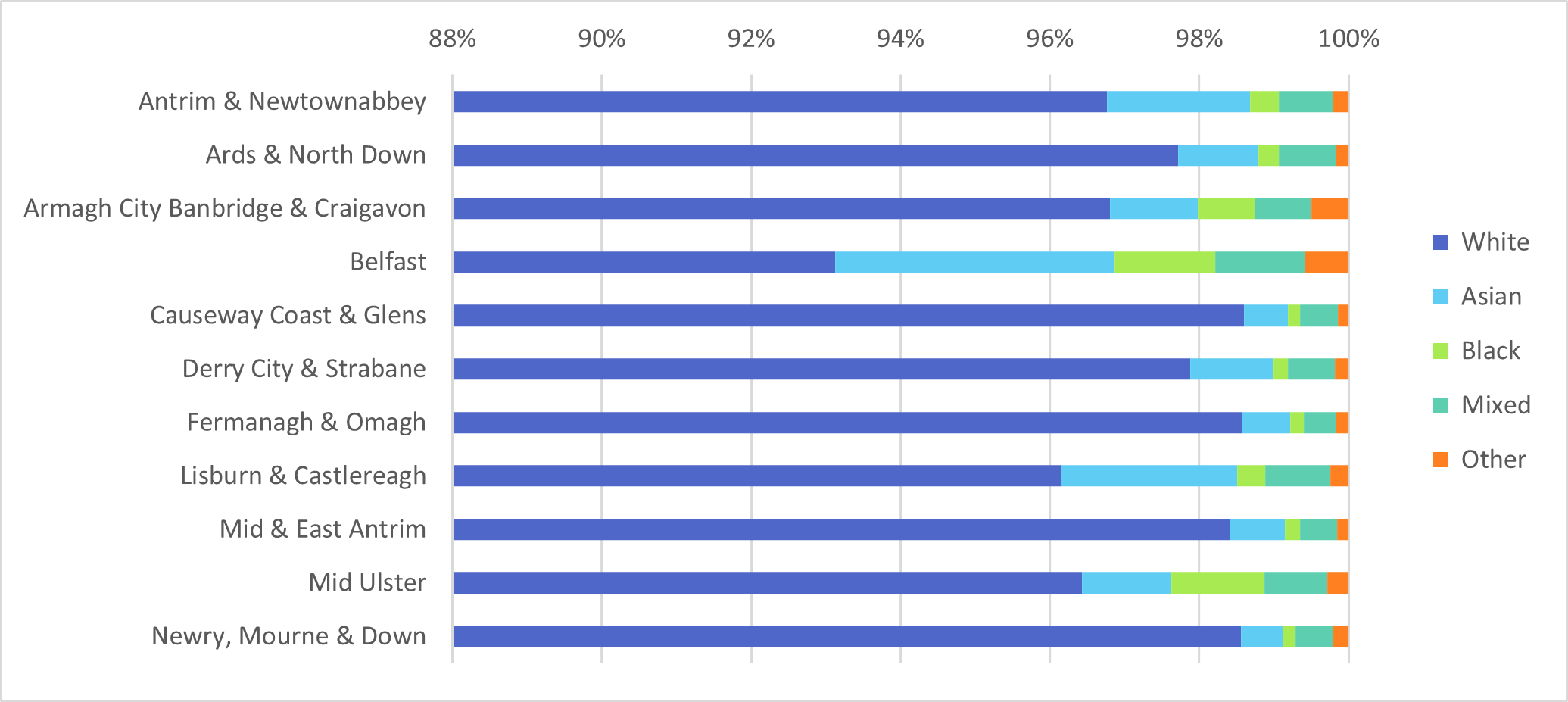

Supplement: Supplementary file 7 — Additional file 7: Fig. S7 Bar graph demonstrating Northern Irish whole population ethnic group breakdown per local government district. [file 13063_2024_8487_MOESM7_ESM.png]

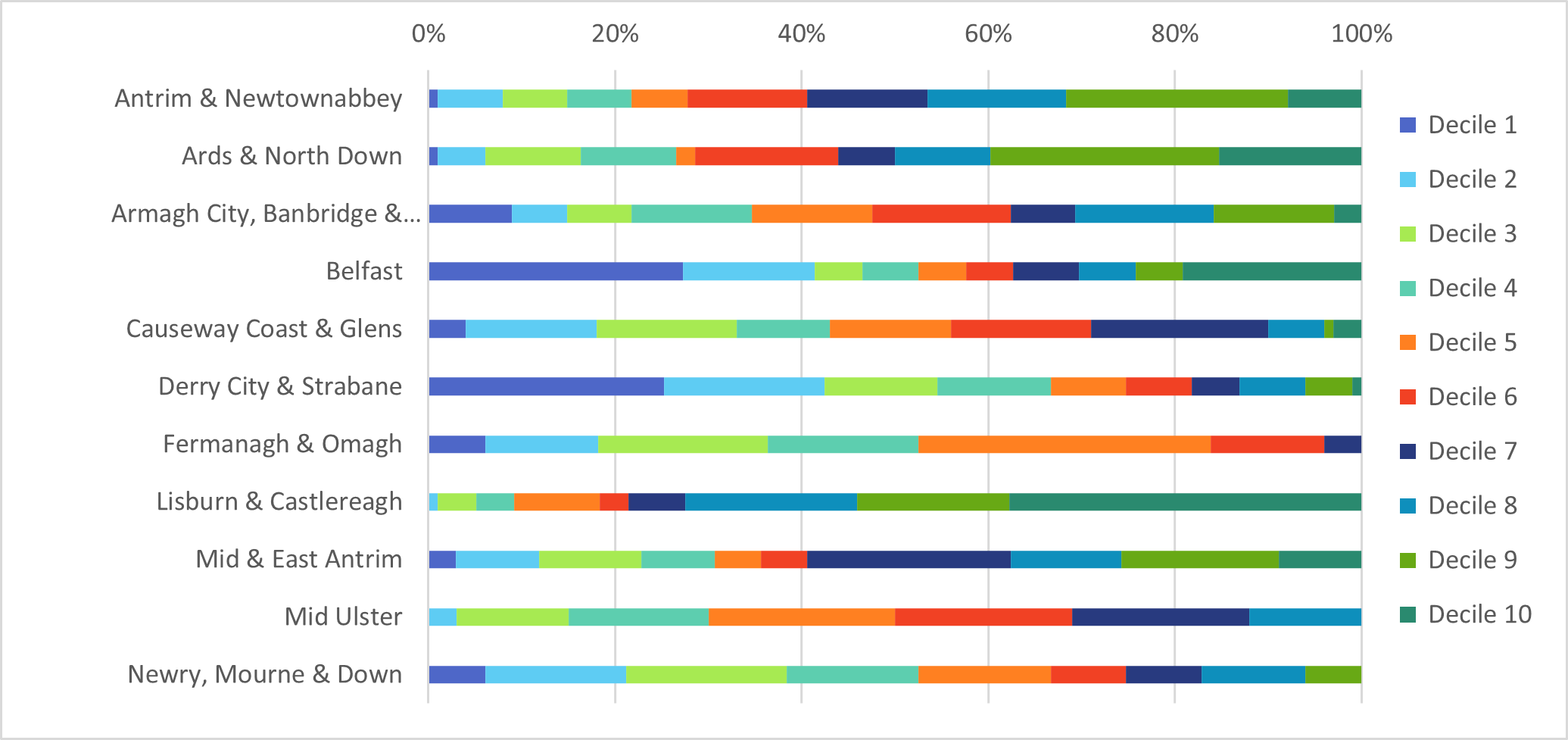

Supplement: Supplementary file 8 — Additional file 8: Fig. S8 Bar graph demonstrating Northern Irish Multiple Deprivation Measure (NIMDM) per local government district. [file 13063_2024_8487_MOESM8_ESM.png]
